# Do delayed responses introduce bias in ecological momentary assessment? Evidence from comparisons between self-reported and objective physical activity

**DOI:** 10.3389/fpsyg.2024.1503411

**Published:** 2025-01-03

**Authors:** Stefan Schneider, Meynard J. Toledo, Doerte U. Junghaenel, Joshua M. Smyth, Pey-Jiuan Lee, Sarah Goldstein, Olivia Pomeroy, Arthur A. Stone

**Affiliations:** ^1^Dornsife Center for Self-Report Science and Center for Economic and Social Research, University of Southern California, Los Angeles, CA, United States; ^2^Department of Psychology, University of Southern California, Los Angeles, CA, United States; ^3^Leonard Davis School of Gerontology, University of Southern California, Los Angeles, CA, United States; ^4^Department of Psychology, The Ohio State University, Columbus, OH, United States

**Keywords:** ecological momentary assessment (EMA), ambulatory assessment (AA), physical activity, response delay, compliance, experience sampling method (ESM)

## Abstract

**Introduction:**

Delayed responses are a common yet often overlooked aspect of participant compliance in ecological momentary assessment (EMA) research. This study investigated whether response delays introduce selection bias in the moments captured by EMA.

**Methods:**

Participants (*n* = 339) self-reported their physical activity behaviors using EMA five times a day over 7 days while wearing a continuous physical activity monitor. The continuous activity monitor data provided an objective reference value to evaluate potential biases in delayed EMA self-reports.

**Results:**

Results showed that participants were significantly more likely to delay EMA responses when they were prompted during higher levels of physical activity, and they subsequently reduced their activity levels, postponing their response until they were in a significantly less active state. There was no significant evidence that response delays systematically biased the levels of EMA reported activities, although delayed responses were associated with significantly more random errors in EMA reports (with small effect sizes).

**Discussion:**

The results suggest that respondents self-select the moments for answering EMA surveys based on their current activity levels, but brief response delays do not translate into marked reductions in the quality of EMA data.

## Introduction

The use of ecological momentary assessment (EMA) methods has substantially risen in popularity in psychological research over the past decades (Stone et al., 2023a). In EMA studies, participants are typically asked to complete brief surveys about their current behaviors, environments, and experiences, several times per day and typically over multiple days (Stone and Shiffman, 1994). Even though this assessment strategy yields rich and granular information about the ebb and flow of people’s behaviors and experiences in everyday life, it is also demanding for participants to respond to all prompts in a timely manner. Over the course of a study, they are expected to briefly but repeatedly interrupt their ongoing daily activities to answer EMA questions when prompted to do so. Moreover, as many EMA studies intentionally signal respondents at (stratified) random times of the day, participants often cannot anticipate the timing of the assessments, and they are asked to respond regardless of whether or not this is currently convenient to them (Shiffman et al., 2008).

In view of this respondent burden, ensuring that participants comply reasonably well with the protocol has been a salient concern in EMA research for many years. To make reliable and valid conclusions from EMA data, it is desirable that the momentary data collected are a representative sample of respondents’ experiences and behaviors in their daily lives (Shiffman et al., 2008). If participants do not adhere to the study protocol and self-select the moments they report on, selection bias (or sampling bias) occurs, because not all situations or experiences have an equal chance of being measured. The most overt form of protocol noncompliance is when participants show poor momentary response rates and miss EMA prompts. Given the perils associated with (especially nonrandom) missing data (Graham, 2012), a sizeable body of EMA research has examined the correlates and consequences of missing data due to skipped EMA assessments (see, for example, Jones et al., 2019; Ono et al., 2019; Vachon et al., 2019; Wrzus and Neubauer, 2023).

### Delayed responding to EMA prompts

Apart from skipping prompts entirely, participants may also engage in more subtle forms of noncompliance, such as delaying responses to EMA prompts (Eisele et al., 2021). In fact, one routinely employed strategy to reduce the amount of missing data due to skipped EMA prompts is to allow participants to temporarily “snooze” prompts or postpone their responses if the signal happens at an inopportune moment; that is, to complete the assessment within a certain time window *after* an EMA prompt occurred (Christensen et al., 2003; Stone et al., 2023a). In a typical EMA study, the delayed assessment is meant to retrospectively collect information about what was happening at the initial prompt. In this manner, the fidelity of the EMA sampling scheme is in theory preserved, because information is collected “as if” there was no delay in the response. If the targeted information is not recovered in delayed reports because respondents do not adequately recall the information from the initial prompt and/or report what was happening after the prompt, then delayed responses may not maintain sampling fidelity.

In most studies, researchers select a maximum delay period after which prompts can either not be answered or after which answers are later discarded from the data and are treated as if they were missed. The duration of the maximum allowed response delays is often constrained to less than 20–30 min (Christensen et al., 2003), even though studies have allowed delays ranging from as little as 1 min (Businelle et al., 2016) to more than 1 h after the occurrence of an EMA prompt (Bekman et al., 2013). Few articles provide an explicit rationale for the selected maximum allowable delay or document the response delay observed in a study (De Vries et al., 2021).

Even though response delays are widely employed and accepted in EMA studies, the potential implications of delayed responding have found limited attention in methodological EMA research (Eisele et al., 2021; Stone et al., 2023a). The concern addressed in this study is whether delayed responding creates a selection bias in the moments captured with EMA. When participants are allowed to postpone their responses, they can (to some degree) self-select the moments during which they complete a given EMA prompt. There are reasons to assume that this may in turn yield a biased representation of people’s daily life experiences. For example, respondents may be more likely to delay prompts when they are currently very active (e.g., when they are exercising), or experiencing strong emotions (e.g., when they are arguing with another person), and they may delay the response until they have returned to a more calm or “normal” state. If respondents report on this more calm or “normal” present state rather than the state at the time of the prompt (either because they follow explicit EMA instructions to report on their experiences “right now” or because they are unable to recall their experiences when EMA items ask them to report what happened “at the prompt”), this could lead to an underrepresentation of very active or emotionally intense moments collected with EMA (Eisele et al., 2021). If this was the case, it could affect measures of frequency, intensity, or duration of behaviors and experiences across individuals in between-person analyses, and it could also distort (e.g., reduce) the magnitude of observed fluctuations within individuals in within-person analyses. To date, little is known about whether delayed responses are merely a harmless departure from the optimal study protocol or if they indeed introduce significant and meaningful selection bias in EMA (Stone et al., 2023a).

### Previous research on EMA response delays

The few studies that have been conducted in this area roughly fall into two categories. The first type of study has focused on identifying potential predictors of EMA response delays. Sarker et al. (2014) examined physiological sensor data collected from 30 university students and found that participants were least likely to delay responding to a prompt when they were walking outside, and most likely to delay at work and when they were driving. Similarly, Boukhechba et al. (2018) found that combinations of passively recorded contextual variables (time of day, location, and phone movement) and participant-phone interactions predicted the length of response delays in 65 university students with moderate accuracy. These results support the idea that momentary behaviors and environmental characteristics are systematically associated with delayed responding in EMA. A strength of these studies is that they analyzed variables that were passively recorded in real time at the time of the delays. At the same time, because the EMA reports themselves were not analyzed, these results do not answer the question whether response delays in fact introduced biases in participants’ self-reported momentary behaviors and experiences.

The second type of study has examined differences in self-reported experiences between prompts to which participants responded immediately and prompts for which they delayed the response. Affleck et al. (1998) compared fibromyalgia patients’ reports of momentary pain, mood, and fatigue between random selections of 100 delayed and 100 immediate responses that were matched by time of day and day of week; they did not find significant differences for either pain, mood, or fatigue reports. In contrast, a large secondary data analysis of over 1,500 individuals from nine different paper-and-pencil EMA studies (Eisele et al., 2021) showed that participants tended to rate their positive affect as significantly higher and negative affect as significantly lower when they had delayed their responses, as compared to immediate EMA responses. Effect sizes were small yet consistent across different (nonclinical and clinical) samples (Eisele et al., 2021). Even though these results suggest that delayed momentary self-reports may be different (e.g., more positive) on average than immediate reports, they do not allow firm conclusions about the extent to which delayed responding introduces selection bias in EMA. If, for example, participants tend to delay responding in situations that are associated with more extreme mood states (e.g., high negative or low positive affect) and respond at their convenience when their mood levels have normalized, the sampled mood levels may not differ between immediate and delayed responses despite (or precisely because of) self-selection. The counterfactual question “what value would a person who delayed a response have provided had they *not* delayed the response” is not directly addressed by comparing EMA ratings between delayed and immediate responses.

### The present study

Taken together, prior literature provides a fragmented picture of the impact of response delays on the representativeness of data collected and does not offer firm evidence-based guidance on the use of response delays in EMA studies. The goal of the present study was to conduct a rigorous analysis addressing the question whether response delays introduce selection biases in EMA. To this end, we examined both passive real-time recordings and momentary self-reports of the same construct, that is, we combined the two approaches that previous studies used to examine response delays in the same analysis. More specifically, we examined pre-existing data from an EMA study in which physical activity behaviors were assessed both continuously with accelerometry and with EMA self-reports over the same time period. To illustrate why it is uniquely beneficial to be able to draw on passive recordings and EMA reports of the same construct in combination, one may think of delayed EMA responding as a process of replacement (“imputation”) of missing values: participants initially omit (i.e., miss) a response when prompted, and then replace (i.e., impute) the omitted response with a new value at a later point in time. A critical question is how well the new (observed) value reflects the value of the originally omitted response. Selection bias occurs when the omitted EMA responses are replaced with delayed EMA responses that differ from the omitted ones. By having passive recordings of the same construct assessed with EMA available for the exact time of the omitted EMA response, we were able to use these passive recordings as a reference point or “benchmark” to evaluate how closely the delayed EMA responses reflected the values of the originally omitted responses.

The specific construct used to investigate whether response delays are associated with selection bias targeted people’s physical activity behaviors. Admittedly, physical activity is merely one type of behavior and, as must be the case, is not necessarily representative of the broad spectrum of behaviors and experiences commonly assessed in clinical EMA studies (Shiffman et al., 2008). However, physical activity patterns in daily life are associated with dynamic changes in many constructs relevant to clinical psychology, including everyday changes in affect (Wen et al., 2018), stress (Do et al., 2021), and cognitive function (Zlatar et al., 2022). Moreover, as noted above, to study potential biases associated with temporarily omitted (i.e., delayed) EMA reports, we required a strategy that allowed comparing subjective EMA reports with continuous measurements of their objective counterparts; the construct of physical activity behaviors lends itself ideally to parallel assessments with EMA and with passive recordings via accelerometry (Stinson et al., 2022).

Several research questions were addressed. The first two questions asked whether objective physical activity levels measured with accelerometry (a) *predicted* the occurrence of response delays, and (b) whether these activity levels *changed* over the course of a response delay; together, this addresses the question whether people tend to systematically self-select the moments at which they complete EMA assessments. The third question asked whether the self-selection of moments of an EMA response translates to biases in *self-reported* activity levels in EMA.

#### Question 1: do momentary physical activity levels predict delayed EMA responding?

Our first analysis focused on objective (i.e., passively recorded) physical activity levels at the time (i.e., during the minute) of a given EMA prompt. Our question was whether physical activity levels at the time of the prompt are systematically associated with the likelihood of delaying a response. We hypothesized that individuals will be less likely to delay EMA prompts when they are more sedentary and more likely to delay EMA prompts when they are more physically active at the time of a prompt, because being currently physically active may make it more difficult and less convenient to immediately respond to an EMA prompt (Dunton, 2017).

#### Question 2: is delayed responding associated with changes in physical activity levels?

Our second question was whether people’s objective physical activity levels *changed* over the course of a response delay, that is, from the time of the EMA prompt to the time of the actual response. Selection biases in EMA are arguably more likely to occur if respondents are objectively in a systematically different state when they respond after a delay compared to their state when they were initially prompted. We hypothesized that individuals will tend to respond after an observed decrease in their physical activity (i.e., from elevated activity levels to a more resting state from the time of a prompt to the time of the delayed response).

#### Question 3: how well do delayed self-reports of physical activity reflect respondents’ objective physical activity levels at the time of the initial EMA prompt?

Our third question was whether the self-selection of moments of an EMA response translates into actual biases in EMA self-reports. Even if the moments at which individuals choose to respond to EMA prompts are self-selected based on current activity levels, this does not necessarily imply that delayed EMA ratings differ from the answers people would have given had they responded right away. To address this question, we compared people’s self-reported physical activity levels with their objective physical activities that were passively recorded at the time of the prompt. This allowed us to examine if delayed self-reports reflected people’s objective physical activity levels at the time of the prompt less well than self-reports that were not delayed. More specifically, we examined if delayed reports introduce (a) systematic biases and (b) random errors in people’s EMA self-reports.

#### Question 3a: are response delays associated with systematic biases in the levels of self-reported physical activity?

When individuals change their physical activity levels from the time of the prompt to the time of a delayed response, their self-reported physical activity levels may be affected by response delays, as well, given that self-reports are impacted by people’s present states (Schneider and Stone, 2016). We hypothesized that response delays would be associated with systematically lower self-reported physical activity levels compared to what would be expected based on objective physical activity levels recorded at the time of the prompt.

#### Question 3b: are response delays associated with more random errors in people’s self-reported momentary physical activities?

Apart from systematic shifts in the levels of people’s self-reports, delaying an EMA response to a later time point may reduce their ability to precisely report their behaviors at the time of the prompt. Memory for momentary experiences and behaviors fades quickly over time (Conner and Barrett, 2012; Walentynowicz et al., 2018), and people may not be fully able to recall their physical activities accurately even with a delay of just a few minutes after an EMA prompt. Accordingly, we hypothesized that self-reported physical activities are less strongly correlated with objective physical activity levels recorded at the time of the prompt when people delayed their EMA responses compared to when EMA responding was not delayed.

## Materials and methods

### Participant sample

The data analyzed for this study came from a larger EMA protocol examining relationships between self-reported and objectively recorded physical activity levels. Study participants were recruited from the Understanding America Study (UAS) (Alattar et al., 2018), a probability-based internet panel study of about 13,000 US residents. Members are recruited to be part of the UAS through address-based sampling, and individuals without prior internet access receive a tablet and broadband access (excluding smartphones). This ensures broad coverage in populations typically underrepresented in opt-in or volunteer online panels. UAS panelists were eligible to participate in the EMA study if they (1) were fluent in English; (2) were 18 years of age or older; (3) had no hearing impairments, (4) had no vision impairments that could not be corrected with contact lenses or glasses; (5) did not work a night shift; (6) had stable access to email; (7) had daily access to Wi-Fi; (8) were not on bed-rest; and (9) did not require any mobility devices to be able to move around. Panelists were contacted via email to be invited into the study and to complete the eligibility screening survey. Of 1,363 UAS panelists who were contacted and assessed for eligibility, 1,021 met the eligibility criteria. Of those, 407 provided consent and were enrolled into the study, and 359 completed the study protocol. The study was approved by the Biomedical Research Alliance of New York Institutional Review Board (#22-183-1044). Participants provided electronic informed consent for participation.

### Study procedures

The study included a 7-day EMA protocol that was executed remotely; participants were mailed a study package to their home that contained the study devices (a smartphone with charger and an activity monitor with medical tape and alcohol swabs) and mailing materials for returning the package after completing the EMA study. The day before starting the protocol, participants were instructed to watch a 20-min training video that explained how to put on and wear the activity monitor, and that guided participants through all aspects of EMA survey completion (how to interact with the device and app, the questions presented, and how to enter their responses). Starting on the next day, participants received five EMA prompts per day for 7 days while continuously wearing their physical activity monitor. Research staff checked the number of completed EMA prompts daily and contacted participants over the telephone and through email if there were suspected technological problems. Participants received email reminders about completing the study activities on a daily basis. Upon study completion, participants returned their study package using the provided mailing materials and prepaid return label. Participants were compensated up to $200 for study participation, which was prorated based on their level of overall protocol completion: participants received $15 for completing the training, $10 for completing the baseline questionnaire, $2 for each EMA survey completed, $10 for each day of wearing the activity monitor, $10 for completing a final questionnaire, and $25 for returning the study devices at the end of the protocol.

### Accelerometry-based physical activity measurement

The activPAL™ activity monitor was used to objectively measure participants’ physical activity levels over the study period. Participants were asked to wear the monitor for seven consecutive days, 24 h a day, except during water-based activities. Unlike hip or wrist-worn accelerometry-based activity monitors, the activPAL is worn on the anterior midline of the thigh. This allows to detect the orientation of the participant’s thigh and to discriminate different postures (upright versus seated or lying down). Upon return of the study device, the raw accelerometer data was downloaded and processed using activPAL’s software (PALanalysis v9.1.0.102). Using the software’s proprietary algorithm (CREA v1.3 algorithm) the raw accelerometry data was processed into events of (a) sedentary (i.e., sitting or lying down), (b) standing, and (c) stepping behaviors (i.e., step counts). Numerous validation studies support the accuracy of the device and algorithm (e.g., Bassett et al., 2014; Lyden et al., 2017). We further used the cadence of momentary stepping activities to distinguish times spent in (d) light physical activity (LPA; cadence < 100 steps/min), (e) moderate physical activity (MPA; cadence 100–125 steps/min), and (f) vigorous physical activity (VPA; cadence > 125 steps/min) levels (Tudor-Locke and Rowe, 2012).

### EMA data collection

As part of the larger study protocol, EMA items were administered using two different reporting periods such that the EMA questions asked participants about their behaviors and experiences either during “the 5 min before the prompt” or during “the 2 h before the prompt”; the reporting period was assigned to participants in a between-person randomized design. EMA prompts were delivered using a stratified random sampling scheme with five prompts that could occur between 6 a.m. and 10 p.m. in the participant’s time zone, and which was stratified such that the sampled reporting periods covered by the EMA questions were at least 15 min apart. For respondents who were asked to report about the 5 min before the prompt, this was accomplished by dividing the waking day into five equal time bins and randomly selecting prompt times within each of these bins (provided a minimum of 20 min between two prompts). For respondents who were asked to report about the 2 h before the prompt, the waking day was divided into equal time bins with a duration slightly longer than 2 h each. From these time bins, a subset of five bins were randomly selected each day for assessment. EMA questions were administered using the movisensXS app on study provided smartphones (Motorola G Power 2021).

Self-reported physical activity levels were assessed with five EMA items asking participants how many minutes they spent (1) sitting or lying down (i.e., sedentary), (2) standing, (3) in LPA, (4) in MPA, and (5) in VPA during the targeted time (i.e., 5 min or 2 h) before the EMA prompt. The EMA questions were designed to closely mirror the physical activity variables derived from the activPAL activity monitor, while also aligning with items used in prior research on the validity of EMA reports (Stinson et al., 2022). Answers were given by entering the number of minutes using an open-ended numeric response format. Two additional EMA items asked participants how “sedentary” and how “physically active” they were during the targeted time (5 min or 2 h) before the prompt, with answers provided using a 5-point verbal rating scale (not at all – extremely). Respondents reported the sleep and wake time for the past night during the first EMA prompt of each day. EMA items on affect, pain, and fatigue were also administered but were not analyzed here.

Participants were allowed to delay their responses to each EMA prompt for up to 15 min. After an initial alarm that lasted for 1 min, reminder alarms lasting 1 min each were administered by the app 5 and 10 min after the first alarm. The prompt expired 15 min after the beginning of the initial alarm and the participant was unable to complete the EMA survey.

### Analysis strategy

Study participants were included in the present analyses if they provided accelerometer data for all 7 study days and for at least 70% of their waking hours during the week. This excluded 20 of the 359 participants, with a resulting analysis sample size of *n* = 339; of those, *n* = 173 answered EMA questions about the 5 min before the prompt and *n* = 166 reported on the 2 h before the EMA prompt.

Response latencies for all EMA prompts were calculated as the difference (in seconds) between the time the prompt was delivered by the app and the time a respondent started the survey. We categorized responses as “delayed” if a participant started the survey more than 1 min after the EMA prompt, that is, following the end of the initial alarm. Conversely, responses were coded as “immediate” if the survey was initiated within 1 min of the EMA prompt, during the period when the initial alarm was still active. This categorization is akin to answering a phone call while it is still ringing.

Our analyses of response delays considered only EMA prompts that were administered during participants’ waking hours. Periods of sleep and accelerometer non-wear time were excluded from the analyses. Sleep periods were determined using both passive sleep detection from accelerometer data (Courtney et al., 2021) and EMA self-reported sleep and wake times; the two sources of information were combined using a weighted average with an algorithm proposed by Thurman et al. (2018).

#### Analyses for question 1: do respondents’ activity levels predict response delays?

Multilevel logistic regression models were used to examine whether objective physical activities recorded by accelerometry during the minute of the EMA prompt predicted the occurrence of delayed EMA responding. The timeliness of an EMA response (immediate versus delayed) served as binary outcome variable. To evaluate the effects of physical activity behaviors at specific intensity levels, we entered the amount of time sedentary (i.e., lying/sitting), time standing, time in LPA, MPA, and VPA individually as predictor variables in separate regression models. In addition, respondents’ step count during the minute of the EMA prompt was examined as a summary measure of overall physical activity intensity and was entered as predictor in a separate model.

For each physical activity variable, the log odds of a delayed response η_*ij*_ for participant *j* at a given measurement occasion *i* were estimated as described by the following multilevel equation:


$$\mathrm{Level}\,1:\eta_{i\,j}=l\,o\,g\,\left(\frac{d\,e\,l\,a\,y_{i\,j}}{1-d\,e\,l\,a\,y_{i\,j}}\right)=\beta_{0\,j}+\beta_{1\,j}\,P\,A_{i\,j}^{\left(W\,C\right)}$$

(1) $$\mathrm{Level}\,2:\beta_{0\,j}=\gamma_{00}+\gamma_{01}\,P\,A_{j}^{\left(M\right)}+u_{0\,j}$$

$$\beta_{1\,j}=\gamma_{10}+u_{1\,j}$$

We distinguished within-person and between-person and effects of physical activity. At level 1, the within-person level, the log odds of delaying a response were predicted from within-person centered physical activity variables $P\,A_{i\,j}^{\left(W\,C\right)}$; this addresses the question whether individuals are more likely to delay an EMA response when they are more physically active (or more sedentary) than they usually are. At level 2, the between-person level, individual differences in the propensity to delay EMA responses over the course of the study were predicted from person mean levels of the physical activity variables $P\,A_{j}^{\left(M\right)}$; this addresses the question whether respondents who are generally more active (or more sedentary) are more likely to delay EMA responses. Also at level 2, the models allowed for residual variance in the random intercepts β_0*j*_ (individual differences in the propensity of response delays not explained by physical activity) and for between-person variance in the regression slopes β_1*j*_ (individual differences in the within-person effect of physical activity on response delays).

To facilitate interpretation of the magnitude of between-person variance in the regression slopes β_1*j*_, we computed the coefficient of variation (CV) as a measure of relative variability. The CV was calculated as the ratio of the standard deviation in regression slopes to the mean regression slope (i.e., the fixed effect, γ_10_). A CV of 1.0 indicates that the regression slopes for most individuals (approximately 68%, assuming normally distributed random effect variances) range between 0 (no effect) and two times the average slope, suggesting substantial heterogeneity in effects.

#### Analyses for question 2: is delayed responding associated with changes in activity?

To investigate whether respondents’ objective activity levels changed from the time of the initial alarm to the time of a delayed response to an EMA prompt, we analyzed the accelerometry data recorded for two time points: (a) the time point (i.e., minute) of the EMA prompt, and (b) the time point (i.e., minute) the participant responded, among those EMA responses that were delayed. Linear multilevel models for change between the two time points were estimated separately for each of the physical activity indicators (amount of time sedentary, standing, time in LPA, MPA, VPA, and step count) as described by the following multilevel equation:


$$\mathrm{Level}\,1:P\,A_{h\,i\,j}^{O\,B\,J}=\beta_{0\,j}+\beta_{1\,j}\,T\,I\,M\,E\,P\,O\,I\,N\,T_{h\,i\,j}+r_{h\,i\,j},$$

$$\text{where}\,r_{h\,i\,j}\,N\,\left(0,\Sigma \right)$$

(2) $$\mathrm{Level}\,2:\beta_{0\,j}=\gamma_{00}+u_{0\,j}$$

$$\beta_{1\,j}=\gamma_{10}+u_{1\,j}$$

The accelerometry-derived measurements taken at the time of the prompt and the time of the response simultaneously served as multivariate (i.e., repeated-measures) dependent variable. $P\,A_{h\,i\,j}^{O\,B\,J}$ represents the objective physical activity measurement for individual *j* and measurement occasion *i* at time point (time of prompt or response) *h* (Snijders and Bosker, 1999). The dichotomous predictor variable *TIMEPOINT* was coded as 0 for measurements taken at the time of the prompt and as 1 for measurements taken at the time of the delayed response. Accordingly, the level 1 intercept β_0*j*_ represents a given respondents’ physical activity level at the time of the prompt (on average across measurement occasions), and the regression slope β_1*j*_ represents the respondent’s change in activity from the alarm to the time of the delayed response (on average across measurement occasions). Random effects for the intercept β_0*j*_ and rate of change β_1*j*_, modeled at level 2, allow for individual differences in both parameters.

To account for nonindependences of activity levels at the time of an EMA prompt and the time of the response within a given measurement occasion, the within-person residuals *r_*hij*_* at level 1 were allowed to correlate through the error variance-covariance matrix Σ. Specifically, an unstructured covariance matrix was used to freely estimate the residual within-person activity variances at the time of the prompt and time of the response, as well as their covariance, across measurement occasions.

#### Analyses for question 3: how well do delayed EMA reports reflect objective activity levels at the time of the prompt?

As described above, participants for this study were randomized to receive one of two different versions of the same EMA items that either asked about “the 5 min before the prompt” or “the 2 h before the prompt”. These subsamples were analyzed separately in the analyses comparing self-reported and objective physical activity levels described below. Because EMA typically employs brief reporting periods, we present primary results based on the subsample of respondents who received the 5-min version of the EMA items, *n* = 173. Secondary results from the subsample of respondents receiving the 2-h version of the items (*n* = 166) are shown in Supplementary material.

#### Analysis for question 3a: are response delays associated with systematic biases in the levels of self-reported physical activity?

To examine whether response delays introduced systematic bias in self-reported activity levels, we compared respondents’ answers on each of the five EMA items (i.e., amount of time sedentary, standing, time in LPA, MPA, and VPA) with respondents’ objective physical activity levels for corresponding variables recorded during the 5 min^1^ before each prompt, and examined whether any activity level differences between these two assessment types were moderated by response delays. In other words, we examined the difference between EMA self-reported and objective measurements as a function of response delay. Linear multilevel models were used for this purpose as described by the following equation:


$$\mathrm{Level}\,1:P\,A_{h\,i\,j}=\beta_{0\,j}+\beta_{1\,j}\,A\,S\,S\,E\,S\,T\,Y\,P\,E_{h\,i\,j}+$$

$$\beta_{2\,j}\,D\,E\,L\,A\,Y_{h\,i\,j}+\beta_{3\,j}\,{\left(A\,S\,S\,E\,S\,T\,Y\,P\,E^{*}\,D\,E\,L\,A\,Y\right)}_{h\,i\,j}+r_{h\,i\,j},$$

where *r*_*ij*_*N*(0,Σ)


(3) $$\mathrm{Level}\,2:\beta_{0\,j}=\gamma_{00}+u_{0\,j}$$

$$\beta_{1\,j}=\gamma_{10}+u_{1\,j}$$

$$\beta_{2\,j}=\gamma_{20}+u_{1\,j}$$

$$\beta_{3\,j}=\gamma_{20}+u_{1\,j}$$

The self-reported and objective measurements of a physical activity variable (time sedentary, standing, LPA, MPA, and VPA; examined in separate models) simultaneously served as multivariate dependent variable. Thus, *PA*_*hij*_ represents the value of a physical activity variable for participant *j* at measurement occasion *i* and assessment type (i.e., self-reported or objective) *h*.

The dichotomous predictor variable *ASSTYPE* was coded as 0 for objective and as 1 for EMA self-reported assessments. The dichotomous predictor variable *DELAY* was coded as 0 for immediate and as 1 for delayed responses. Both predictors were within-person centered before computing the *ASSTYPE* × *DELAY* interaction term so that they can be interpreted analogous to within-person main effects (Aiken et al., 1991; Yaremych et al., 2023). Accordingly, the regression slope β_1*j*_ represents the difference between EMA self-reported and objective activity levels, and the regression slope β_2*j*_ represents the difference in activity levels between prompts that were delayed versus not delayed. The interaction term β_3*j*_ compares self-reported and objective activity levels between immediate and delayed responses, testing the hypothesized effect. If the interaction term is significant, this indicates that the self-reported activity levels for delayed EMA responses differ from (i.e., are systematically higher or lower than) what would be expected had respondents answered the prompts immediately.

Level 2 random effects for the intercept β_0*j*_ and regression parameters β_1*j*_, β_2*j*_, and β_3*j*_ allowed for individual differences in each of these parameters. To account for nonindependences of a person’s self-reported and objectively recorded activity levels at the same measurement occasion, the within-person residuals *r*_*hij*_ at level 1 were allowed to correlate through an unstructured variance-covariance matrix Σ.

#### Analysis for question 3b: are response delays associated with more random errors in self-reported physical activities?

Finally, to examine whether response delays introduced unsystematic (“random”) errors in respondents’ EMA reports, we examined whether the magnitude of associations between self-reported and objective activity levels was moderated by response delays. The following multilevel model was estimated for this purpose:


Level⁢1:P⁢Ai⁢jO⁢B⁢J=β0⁢j+β1⁢j⁢P⁢AE⁢M⁢Ai⁢j+β2⁢j⁢D⁢E⁢L⁢A⁢Yi⁢j+

$$\beta_{3\,j}\,{\left(P\,A^{E\,M\,A}\,\text{×}\,D\,E\,L\,A\,Y\right)}_{i\,j}+r_{i\,j}$$

where *r*_*ij*_*N*(0,σ^2^)


(4) $$\mathrm{Level}\,2:\beta_{0\,j}=\gamma_{00}+u_{0\,j}$$

$$\beta_{1\,j}=\gamma_{10}+u_{1\,j}$$

$$\beta_{2\,j}=\gamma_{20}$$

$$\beta_{3\,j}=\gamma_{20}$$

Here, $P\,A_{i\,j}^{O\,B\,J}$ represents the objective physical activity variable (time sedentary, standing, LPA, MPA, and VPA; examined in separate models) recorded over the 5 min before the prompt for occasion *i* and person *j*. At level 1, it was regressed on the within-subject centered EMA self-report item for the same physical activity, *PA^EMA^*, a within-subject centered binary indicator for delayed versus immediate responses, *DELAY*_*ij*_, and the EMA self-report by response delay interaction, *PA^EMA^* × *DELAY*_*ij*_. Accordingly, the regression slope β_1*j*_ represents the magnitude of the within-subject association between self-reported and objective physical activities, and the regression slope β_2*j*_ represents the difference in objective activity levels between prompts that were delayed versus not delayed. The interaction term β_3*j*_ indicates whether the magnitude of a within-subject association between self-reported and objective physical activities is moderated by delayed responses, testing the hypothesized effect. If the interaction is significant, this indicates that the association differs between responses that were delayed versus not delayed.

At level 2, the intercepts β_1_*_*j*_* and regression slopes of the EMA self-reports β_1_*_*j*_* were modeled as random effects. The remaining parameters β_2_*_*j*_* and β_3_*_*j*_* were not modeled as random effects because the between-person variances for these parameters were found to be zero in analyses after controlling for the fixed effect parameters.

In addition to the five EMA items that asked participants how many minutes they spent at different physical activity levels using an open-ended numeric response format, two EMA items that used a 5-point verbal rating scale format (“how sedentary were you?” and “how physically active were you?”; not at all – extremely) were also analyzed here to examine whether the strength of their association with corresponding objective physical activity variables (time sedentary; time in light or higher activity levels) was moderated by response delays.

This study’s design and its analysis were not preregistered. We report fit statistics, including −2 × log likelihood, AIC, and BIC, alongside the parameter estimates to provide a transparent overview of model performance. These metrics quantify the balance between model fit and complexity. We did not conduct fit comparisons with more restrictive (error) variance structures; we freely estimated random effects for all model parameters unless this led to non-positive definite covariance matrices, to account for potential heterogeneity in the estimated effects across individuals. All data, analysis code, and research materials are available upon request from the first author. Data were analyzed using SAS version 9.4 (Cary, NC, USA).

### Sample size and statistical power

Statistical power in multilevel models depends on a series of factors, including sample size, number of repeated observations, the between- and within-person variance composition, and the magnitude of random effects variances (Bolger and Laurenceau, 2013). Because research questions 3a and 3b involved lower sample sizes, we estimated the smallest effect sizes detectable with a sample of *n* = 173 and 30 observations per person (i.e., assuming 15% missing data for five daily EMA prompts over 7 days) for these two questions. Power calculations were conducted using Monte Carlo simulations consisting of 1,000 replications in M*plus* version 8.10 (Muthén and Muthén, 2017). Based on prior EMA studies (Ono et al., 2019; Podsakoff et al., 2019) we assumed 15% delayed responses, a ratio of random intercept to within-person variances of 0.5/1, and a ratio of random intercept to random slope variances of 1/0.2 for all random effects. For question 3a, the analyzed sample provided 80% power to detect an effect size of 0.27 (α = 0.05) for the two-way within-person interaction (standardized difference in differences) between assessment type (EMA versus objective recordings) and response delay (immediate versus delayed responses). For question 3b, the sample provided 80% power to detect differences in standardized within-person regression coefficients between immediate and delayed responses as small as β = 0.035 (α = 0.05), assuming a regression coefficient of 0.50 for the relationship between objectively recorded and EMA reported activities for immediate responses.

## Results

Demographic characteristics of the study sample are shown in Table 1. Participants’ mean age was 48.83 years (SD = 14.91; range = 19–86 years). About half (51.78%) were female, four fifths (79.88%) were White, one seventh (14.79%) were Hispanic, and about three fourths (72.19%) were married. The majority (65.68%) had graduated college.

**TABLE 1:** Demographic characteristics of the study sample (*N* = 339).

	Frequency (%) or mean (SD)
Age (mean, SD)	48.84 (14.89)
Female	176 (51.92%)
**Race**	
White	271 (79.94%)
Black	22 (6.49%)
American Indian or Alaska Native	5 (1.47%)
Asian	17 (5.01%)
Native Hawaiian or Pacific Islander	3 (0.88%)
Mixed	21 (6.19%)
Hispanic	50 (14.75%)
**Education**	
Less than high school	8 (2.36%)
High school graduate	42 (12.39%)
Some college	66 (19.47%)
College graduate	35 (10.32%)
Bachelor’s degree	98 (28.91%)
Master’s degree or higher	90 (26.55%)
**Income^a^**	
Less than $30,000	45 (13.31%)
30,000–$49,999	42 (12.43%)
50,000–$99,999	105 (31.07%)
100,000-$149,999	57 (16.86%)
150,000 or more	89 (26.33%)
**Marital status**	
Married	245 (72.27%)
Separated/divorced	32 (9.44%)
Widowed	7 (2.06%)
Never married	55 (16.22%)

*^a^*Income was not reported by one participant.

**FIGURE 1:**
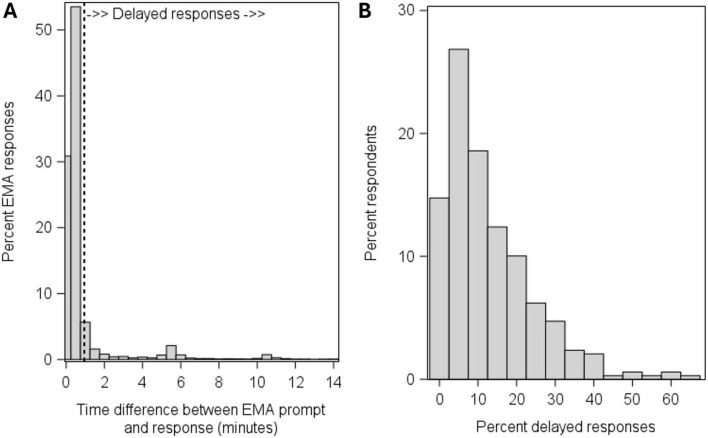
Distribution of response latencies for individual EMA prompts **(A)** and distribution of the percent delayed responses across participants **(B)**.

The distribution of response latencies in minutes for all individual EMA prompts is shown in Figure 1A. Out of 11,270 prompts, 9,125 (80.97%) were answered within 1 min of the prompt (i.e., before the initial alarm had ended) and were coded as immediate responses, 1,225 (10.87%) were answered more than 1 min after the prompt and were coded as delayed responses; the remaining 920 (8.16%) prompts were missed. Figure 1B shows the between-person distribution in the number (i.e., proportion of prompts) of delayed responses; the interquartile range of delayed responses was between 3.1% and 18.8% across participants. To descriptively examine the extent to which response delays were attributable to stable between-person or to momentary within-person factors, we calculated the intraclass correlation coefficient (ICC) for the occurrence of response delays.^2^ The ICC was 0.25, indicating that about 25% of the variation in response delays was due to between-person differences, whereas the remaining 75% was attributable to moment- or situation-specific factors.

### Do objective activity levels predict EMA response delays?

As shown in Table 2, within-person effects of current physical activity levels predicting the occurrence of delayed responses were highly significant. The probability of delaying a response decreased to the extent that a respondent was more sedentary than usual, and increased when a respondent spent more time than usual standing, in LPA, MPA, and VPA. Specifically, the odds of delaying a response decreased by 10% for every 10 s a participant spent more time in sedentary states during the minute of the prompt, and increased by 9%, 24%, 13%, and 55% for every 10 s a participant spent more time standing, in LPA, MPA, and VPA, respectively. The effects of time spent sedentary and standing showed substantial variation between individuals: the between-person standard deviation of regression slopes was close to (CV = 0.95 for sedentary) or greater than (CV = 1.36 for standing) the mean regression slope for these variables. In contrast, the variability in the effects of LPA, MPA, VPA, and step counts was less pronounced (CV = 0.05–0.30).

**TABLE 2:** Results from multilevel logistic regression models predicting the occurrence of delayed responses from objective measures of physical activity recorded during the minute of the EMA prompt.

Parameter	Sedentary	Standing	LPA	MPA	VPA	Steps
**Fixed effects**						
Intercept	−1.694 (0.28)***	−2.361 (0.14)***	−2.449 (0.12)***	−2.260 (0.08)***	−2.201 (0.07)***	−2.458 (0.12)***
Within-person	−0.110 (0.01)*** OR = 0.90	0.087 (0.02)*** OR = 1.09	0.213 (0.03)*** OR = 1.24	0.118 (0.05)* OR = 1.13	0.438 (0.15)** OR = 1.55	0.128 (0.02)*** OR = 1.14
Between-person	−0.135 (0.07)* OR = 0.87	0.105 (0.09) OR = 1.11	0.520 (0.22)* OR = 1.68	0.631 (0.42) OR = 1.88	1.499 (1.57) OR = 4.48	0.325 (0.13)* OR = 1.38
**Variance components**						
Intercept	0.828	0.822	0.816	0.822	0.825	0.827
Within-person regression slope	0.011	0.014	0.004	0.000	0.000	0.001
Covariance	0.002	0.007	0.015	−0.001	0.000	0.010
**Fit statistics**						
−2 log likelihood	49,610.05	49,457.42	49,709.94	49,532.11	49,532.13	49,693.81
Generalized χ^2^	7,430.02	7,481.55	7,743.55	7,747.98	7,747.36	7,717.34
χ^2^/df	0.78	0.78	0.81	0.81	0.81	0.81

Standard errors are in parentheses.

*p < 0.05;

**p < 0.01;

***p < 0.001.

Predictors are scaled in 10 s/min (for time sedentary, time standing, time in LPA, time in MVPA) or in 10 steps/min (for step count). LPA, light physical activity; MPA, moderate physical activity; VPA, vigorous physical activity; OR, odds ratio; df, degrees of freedom.

Between-person effects of physical activity mirrored the pattern observed in the within-person effects: respondents who showed more sedentary behaviors in general were significantly less likely to delay EMA responses over the course of the study, and respondents who spent more time in physically active behaviors were more likely to delay responses, even though the latter effects were only statistically significant for time in LPA (see Table 2).

**FIGURE 2:**
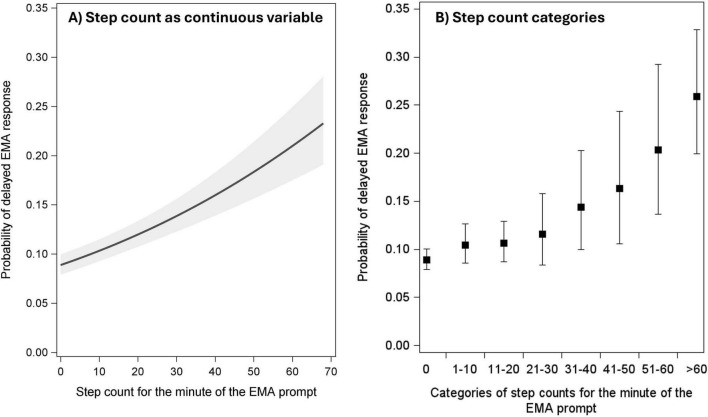
Probabilities of delayed EMA responses by step counts as a continuous variable **(A)** and by step count categories **(B)**. Solid lines and filled circles represent point estimates. Shaded areas and error bars represent 95% confidence intervals.

Corroborating these results, the probability of delayed responses also significantly increased with greater step counts at both within- and between-person levels of analysis (see Table 2). Figure 2A shows the linear effect of step counts as a continuous predictor variable. As it is also plausible that the effects of physical activity are nonlinear or discontinuous (e.g., physical activity levels might need to surpass a certain “threshold” to affect participants’ response behaviors), in secondary analyses, we divided the continuous step count variable into categories (0 steps, 1–10 steps, 11–20 steps, 21–30 steps, etc., during the minute of the prompt) and entered the resulting variable as a categorical predictor of response delays in a multilevel logistic regression model. Figure 2B shows the resulting estimated probability of delayed responses by step count category; the probability of delaying a response was 0.089 for when 0 steps were taken, and it monotonically increased for higher step count categories to a probability of 0.259 when 60 or more steps were taken during the minute of the EMA prompt.

We also explored the question whether physical activities occurring during the minutes *before* an EMA prompt would predict the likelihood of EMA response delays. To examine this, we examined the step counts recorded at increasing time lags (1 min, 2 min, etc., up to 5 min) before the EMA prompt. As shown in Table 3, when the step counts for each of these time lags were entered individually as predictors of response delays in separate multilevel logistic regressions, the effects remained significant for all time lags even though the effect sizes (odds ratios) decreased quickly from OR = 1.14 (for the minute of the prompt) to OR = 1.10 (1 min before the prompt) to OR = 1.05 (4 and 5 min before the prompt). When the step counts for different time lags were entered together as predictors in the same model, only the effect of step counts during the minute of the prompt remained significant (Table 3).

**TABLE 3:** Results from multilevel logistic regression models predicting the occurrence of delayed responses from step counts recorded at increasing time lags before the EMA prompt.

	Odds ratio (95% CI)	
**Predictors**	**Predictors entered individually in separate models**	**Predictors entered together in the same model**
Step count during the minute of the EMA prompt	1.14 (1.10, 1.18)	1.12 (1.07, 1.17)
Step count 1 min before the EMA prompt	1.10 (1.07, 1.13)	1.02 (0.97, 1.07)
Step count 2 min before the EMA prompt	1.09 (1.05, 1.12)	1.02 (0.96, 1.07)
Step count 3 min before the EMA prompt	1.07 (1.04, 1.11)	1.02 (0.97, 1.08)
Step count 4 min before the EMA prompt	1.05 (1.02, 1.08)	0.97 (0.92, 1.03)
Step count 5 min before the EMA prompt	1.05 (1.01, 1.08)	1.00 (0.95, 1.05)

Odds ratios for which the 95% CI does not include 1.0 are significant at *p* < 0.05. CI, confidence interval.

In supplemental analyses, we also examined whether physical activity levels predict the likelihood of missing (i.e., skipped) EMA responses. As EMA prompts expired after 15 min, response delays exceeding 15 min were treated as missed prompts. Paralleling the results for delayed responses, the probability of missing an EMA response significantly decreased to the extent that a respondent was more sedentary than usual, and missed EMA responses became significantly more likely when respondents spent more time in LPA, MPA, and VPA (see Supplementary Table 1).

Alternatively, it is also possible to conceptualize response delays as a time-to-event (i.e., duration) variable, that is, as the time lag from the EMA prompt to when the response occurred (see Elmer et al., in press). Results from time-to-event analyses using Cox proportional hazards models, which examined whether physical activity levels predicted the time to an EMA response, are presented in Supplementary Table 2. Missing responses were right censored, and random effects (frailty terms) were included to account for the clustered nature of the data. Results from the time-to-event analyses were consistent with the primary models, showing that sedentary behaviors predicted a higher probability (i.e., hazard) of responding to EMA prompts earlier, and spending more time in LPA, MPA, and VPA each predicted a lower probability of responding to EMA prompts earlier.

### Is delayed responding associated with changes in objective activity levels?

Results from multilevel models to estimate changes in respondents’ physical activity levels from the minute of an EMA prompt to the minute of a delayed response are shown in Table 4. There was no significant change in the amount of time standing from the time of the prompt to the time of the response. However, participants’ time in sedentary states significantly increased from the time of the EMA prompt to the time of a delayed response. Moreover, participants exhibited significantly lower time in LPA, in MPA, in VPA, as well as a lower number of steps, comparing the minute of the prompt to the minute of the response during a response delay. The random coefficients for change in Table 4 indicate substantial individual differences in the magnitude of these changes between individuals, with CVs ranging from 0.99 for step counts to 3.90 for time in sedentary states (except for VPA, CV = 0.26).

**TABLE 4:** Change in objective physical activity levels from the minute of the prompt to the minute of the response for delayed EMA responses.

Parameter	Sedentary (seconds)	Standing (seconds)	LPA (seconds)	MPA (seconds)	VPA (seconds)	Steps (count)
**Fixed effects**						
Intercept (time of prompt)	32.20 (0.92)***	18.94 (0.76)***	7.09 (0.43)***	1.41 (0.22)***	0.26 (0.11)*	11.76 (0.71)**
Change from prompt to response	2.37 (1.01)*	0.56 (0.90)	−2.28 (0.48)***	−0.44 (0.22)*	−0.24(0.11)*	−4.21 (0.77)***
**Variance components**						
Level 2 random effects						
Intercept	59.95	22.30	4.64	1.60	0.05	26.04
Change	85.36	49.69	7.74	0.65	0.00	17.56
Covariance	25.49	8.49	2.92	0.64	0.02	13.87
Level 1 residuals						
Time of prompt	677.40	527.44	85.24	27.51	0.02	219.25
Time or response	744.11	522.45	180.87	44.14	13.41	502.63
Covariance	−306.95	−197.86	−25.34	−6.85	−0.03	−55.24
**Fit statistics**						
−2 log likelihood	20,998.0	20,329.7	17,187.7	14,324.0	14,506.7	19,409.5
AIC	21,010.0	20,341.7	17,199.7	14,336.0	14,516.7	19,421.5
BIC	21,032.0	20,363.7	17,221.7	14,358.0	14,535.0	19,443.5

Standard errors are in parentheses.

*p < 0.05;

**p < 0.01;

***p < 0.001.

LPA, light physical activity; MPA, moderate physical activity; VPA, vigorous physical activity; AIC, Akaike information criterion; BIC, Bayesian information criterion.

**FIGURE 3:**
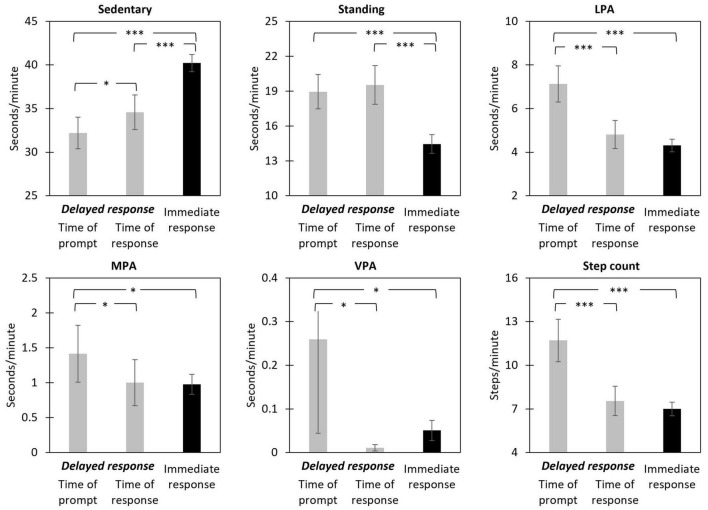
Estimated means of objective physical activity levels during the minute of the EMA prompt and minute of the response (for delayed responses) and during the minute of the EMA prompt (for immediate responses). Means represent seconds per minute in each activity category (for sedentary, standing, LPA, MPA, and VPA) or number of steps per minute (for step count). Error bars represent 95% confidence intervals. LPA, light physical activity; MPA, moderate physical activity; VPA, vigorous physical activity. *p < 0.05; ***p < 0.001 for differences in means.

To put the magnitude of the changes in physical activity levels into context, in secondary analyses, we also explored how these changes compared to respondents’ activity levels for prompts that were *not* delayed. To do this, we expanded the multilevel models for change by adding a categorical indicator representing immediate EMA responses as predictor variable. Figure 3 shows the mean levels for each physical activity variable (a) at the time of the prompt (for delayed responses), (b) at the time of the response (for delayed responses), and (c) for immediate (i.e., nondelayed) responses to EMA prompts. As can be seen, compared to immediate responses, participants spent significantly less time in sedentary behaviors and more time standing at the time of a delayed response. However, for LPA, MPA, VPA, and step counts, the reduction in participants’ activity levels during a response delay occurred to a level that was not significantly different on average from their activity levels for prompts that were answered immediately.

In supplemental moderator analyses, we also explored whether the magnitude of changes in activity levels from the time of the prompt to the time of a delayed response differed by the length of a delay (i.e., by the specific time difference between prompt and response). The specific delay duration did not significantly moderate the changes for any of the activity indicators (see Supplementary Table 3).

### Are response delays associated with systematic biases in self-reported activity levels?

Whether delayed responding translated into biases in EMA self-reports was examined next by comparing self-reported and objective physical activity levels across delayed and immediate responses. We first present the results for the primary analyses, that is, for respondents who were asked to rate their activities for the 5 min before the prompt. As shown in Table 5, the estimated mean activity levels differed significantly between EMA reported and objective assessment types controlling for response delays; participants overall self-reported less sedentary time and more time standing and in LPA, MPA, and VPA, compared to objective recordings of these activities over the same time period. However, the assessment type by response delay interaction term was not significant for the variables examining sedentary time, standing, LPA, and VPA, indicating that the mean difference between EMA reported and objective activities did not significantly differ between immediate and delayed responses for these variables. Notably, there was substantial between-person variability in the magnitude and direction of the interaction terms. For MPA, the interaction term was significant, but the direction of the effect was contrary to the hypothesized effect: self-report ratings of MPA exceeded the objectively recorded MPA more when EMA responses were delayed compared to immediate responses, contrary to our hypothesis.

**TABLE 5:** Results of multilevel models comparing objective and EMA reported physical activity levels for the 5 min before the prompt across immediate and delayed responses.

Parameter (scaled in seconds per minute)	Sedentary	Standing	LPA	MPA	VPA
**Fixed effects**					
Intercept	38.54 (0.58)***	15.25 (0.49)***	11.83 (0.31)***	1.79 (0.12)***	.65 (0.10)***
Assessment type	−4.13 (0.49)***	2.51 (0.63)***	14.41 (0.52)***	1.73 (0.23)***	1.21 (0.21)***
Response delay	−5.18 (1.07)***	3.40 (0.84)***	1.75 (0.64)**	1.26 (0.52)*	.41 (0.19)*
Assessment type × delay	−0.37 (1.01)	0.51 (1.00)	0.63 (1.04)	0.51 (0.30)*	0.55 (0.36)
**Variance components**					
Level 2 random effects					
Intercept	41.58	29.77	10.25	1.21	1.38
Assessment type	27.75	56.30	29.41	5.47	5.68
Response delay	4.36	1.10	1.24	0.00^a^	0.42
Assessment type × delay	17.49	15.39	0.11	0.00^a^	0.72
**Level 1 residuals**					
Accelerometry assessments	565.25	362.83	62.50	25.81	2.14
EMA assessments	629.35	470.34	543.66	118.82	55.15
Covariance	415.97	231.20	58.81	10.20	1.00
**Fit statistics**					
−2 log likelihood	90,814.5	88,916.5	82,290.3	70,469.0	54,829.8
AIC	90,828.5	88,928.5	82,302.3	70,479.0	54,843.8
BIC	90,850.6	88,947.4	82,321.2	70,494.7	54,865.9

*p < 0.05;

**p < 0.01;

***p < 0.001.

Assessment type = EMA reports versus objective recordings. Response delay = delayed versus immediate EMA responses. LPA, light physical activity; MPA, moderate physical activity; VPA, vigorous physical activity.

*^a^*Variance components were fixed at zero to achieve a positive definite covariance matrix of the random effects.

Similar results were found in secondary analyses of the group of respondents who completed EMA items asking about their activities over the 2 h before the prompt (see Supplementary Table 4). Participants self-reported less sedentary time and more time standing, in LPA, MPA, and VPA, compared to objective recordings of these activities over the 2-h period before the prompt. However, the assessment type by response delay interaction term was not significant for any of the physical activity variables, indicating that the differences in the levels of self-reported and objectively recorded activities did not differ according to whether a prompt was immediate or delayed.

### Are response delays associated with more random errors in self-reported activities?

Finally, we examined whether delayed responding moderated the magnitude of associations between self-reported and objective physical activities. We start with results for respondents completing the 5-min version of the EMA items. As shown in Table 6, the results were similar for all physical activity variables except for VPA. For each variable, the regression coefficients of within-subject centered EMA reports predicting objective measurements of corresponding physical activities were highly significant and positive, indicating that self-reported and objective physical activities were positively associated. However, with the exception of VPA, this association was significantly moderated by response delays; the negative interaction term indicated that the association between EMA reported and objective activities was lower for delayed compared to immediate responses. The same pattern of effects was evident for EMA items presented with an open-ended numeric response format and for EMA items using a “traditional” verbal rating scale.

**TABLE 6:** Multilevel regression models predicting objective physical activity levels during the 5 min before the EMA prompt from EMA reported physical activity levels, for immediate versus delayed EMA responses.

	Physical activity outcomes for EMA items with open numeric response format					Physical activity outcomes for EMA items with rating scale format^a^	
**Parameter (scaled in seconds per minute)**	**Sedentary**	**Standing**	**LPA**	**MPA**	**VPA^b^**	**Sedentary**	**LMVPA**
**Fixed effects**							
Intercept	40.34 (0.67)***	13.82 (0.52)***	4.58 (0.19)***	0.94 (0.09)***	0.50 (0.32)	40.87 (0.66)***	5.41 (0.22)***
Delay	−1.11 (0.82)	1.99 (0.76)**	1.12 (0.36)**	0.07 (0.22)	0.03 (0.05)	−1.44 (0.92)	0.03 (0.39)
EMA	0.65 (0.02)***	0.50 (0.02)***	0.12 (0.01)***	0.08 (0.02)***	0.06 (0.05)	10.55 (0.36)***	5.44 (0.26)***
Delay × EMA	−0.07 (0.03)*	−0.11 (0.04)**	−0.04 (0.02)*	−0.08 (0.02)***	0.01 (0.01)	−1.27 (0.65)*	0.90 (0.40)*
**Random effects**							
Intercept	67.10	37.73	4.11	0.76	17.90	62.54	6.30
EMA regression slope	0.06	0.04	0.01	0.04	0.36	15.18	8.39
Covariance	−0.69	0.38	0.05	0.15	2.53	−12.62	5.56
Level 1 residual	255.73	229.27	51.30	20.12	0.77	323.43	58.96
**Fit statistics**							
−2 log likelihood	40,786.3	40,304.7	33,065.7	28,746.9	13,696.0	42,226.1	34,377.2
AIC	40,794.3	40,312.7	33,073.7	28,754.9	13,704.0	42,234.1	34,385.2
BIC	40,806.9	40,325.3	33,086.3	28,767.5	13,716.6	42,246.7	34,397.9

*p < 0.05;

**p < 0.01;

***p < 0.001.

LPA, light physical activity; MPA, moderate physical activity; VPA, vigorous physical activity; LMVPA, light, moderate, or vigorous physical activity.

*^a^*EMA rating scale items were “during the 5 min before the prompt, how sedentary were you?” and “during the 5 min before the prompt, how physically active were you?”

*^b^*Accelerometry and EMA data for VPA were log transformed to facilitate model convergence.

To illustrate the magnitude of the moderated effects, Table 7 shows the within-person correlations between EMA reported and objective activities for each variable, separately for immediate and delayed responses. The correlations ranged between *r* = 0.04 (for time in VPA) to *r* = 0.70 (time sedentary) for immediate responses and between *r* = 0.00 (time in MPA) to *r* = 0.65 (time sedentary) for delayed responses. Notably, delayed reports of MPA showed virtually no correlation with participants’ objective MPA at the time of the prompt, suggesting that these reports were no more informative about objective MPA than random responses. Effect sizes for differences in the Fisher z-transformed correlations (i.e., Cohen’s *q*) between immediate versus delayed responses are also shown in Table 7, where values of *q* = 0.10, 0.30, and 0.50 suggest small, medium, and large effects, respectively (Cohen, 1988). Effect sizes ranged between *q* = 0.08 to *q* = 0.22 (except for VPA; *q* = 0.06), suggesting overall small effects. The negative effect size for VPA indicates that, contrary to expectation, delayed responses were somewhat more highly correlated with actual behaviors compared to immediate responses, though this difference was not statistically significant.

**TABLE 7:** Within-subject correlations between objective physical activity levels during the 5 min before the EMA prompt and EMA reported physical activity levels, for immediate and delayed EMA responses.

	Correlation between objective and EMA self-reported activities		Difference in correlations
**Physical activity variable**	**Immediate response**	**Delayed response**	**Effect size *q***
Time sedentary	0.70	0.65	0.09
Time standing	0.57	0.46	0.15
Time in LPA	0.33	0.23	0.11
Time in MPA	0.21	0.00	0.21
Time in VPA	0.04	0.10	−0.06
Rating of how sedentary	0.65	0.57	0.13
Rating of how physically active	0.54	0.49	0.07

In secondary analyses of respondents who reported their activities over the 2 h before the prompt, response delays did not significantly moderate the association between self-reported and objective activities for most variables except for LPA (see Supplementary Table 5). For time spent in LPA, the within-person correlation between self-reported and objectively recorded activities was *r* = 0.38 for immediate and *r* = 0.27 for delayed responses (*q* = 0.12 for the difference in these correlations, see Supplementary Table 6).

## Discussion

A major advantage of EMA is the ability to collect information about experiences and behaviors in (near) real time. For the methodology to provide optimal results, participants must provide timely EMA reports when prompted. Delayed EMA responses create a temporal misalignment between the occurrence of a behavior and its reporting. However, to date, the conditions generating the misalignment and the consequences of the temporal misalignment for the quality of EMA reports are poorly understood. Using continuous activity monitoring as an objective reference value, we found that participants systematically self-selected the moments when they answered EMA surveys based on their current physical activity behaviors. There was only very limited evidence to suggest that this self-selection of the moments of reporting translated into biases in EMA reports, although random error was increased in delayed reports.

We found that participants on average delayed about 10% of EMA reports by more than 1 min. The likelihood of delays was lower when individuals spent more time in sedentary states and increased to rates of 20% or more for higher activity levels. This finding extends prior literature documenting that contextual and behavioral factors beyond structural barriers to EMA reporting (such as driving, sleeping) can contribute to EMA response delays (Sarker et al., 2014; Boukhechba et al., 2018), and that participants are more likely to skip prompts entirely when they are more physically active (Dunton et al., 2012). It is noteworthy that even small elevations of activity levels (time standing, time in LPA) above sedentary behaviors predicted a greater chance of response delays, suggesting that delays were not exclusively attributable to vigorous physical exercise (e.g., jogging, running). Importantly, both within-person fluctuations and between-person differences in objective physical activity levels contributed in a similar way to the occurrence of delayed responding. It has often been pointed out that failing to distinguish within- and between-person effects can result in “uninterpretable blends” between two possibly very different underlying mechanisms (e.g., Hamaker, 2012). Our results suggest that physical activity monitoring with wearable devices is comparably relevant for understanding and predicting participants who will more likely engage in delayed responding and for predicting the moments and EMA prompts for which a given person will more likely delay a response.

Although the majority of EMA research relies on stratified random prompts to collect self-report data that is most representative of all waking hours of a participant’s day, event-contingent EMA prompts have been used to gain insights into people’s experiences in specific contexts. Increasingly, researchers have leveraged passive activity sensors to trigger event-contingent EMA prompts in “context-aware” sampling designs (Aminikhanghahi et al., 2019; Hoemann et al., 2020). For example, Dunton et al. (2014) proposed using motion sensors integrated in smartphones to detect likely bouts of sedentary and physically active behaviors, and to use times between these bouts to trigger EMA surveys to gather specific information about where, with whom, and why these physical activity states occurred. Context-aware delivery of EMA prompts triggered by activity sensors has also been explored in attempts to limit participant burden, increase response rates, and facilitate timely EMA responses (Khanshan et al., 2021; King et al., 2024). Our results indicated that higher activity levels (greater step counts) monotonically increased the chances of response delays, but they did not reveal a threshold of activity levels that would yield categorically different response behaviors. Moreover, even though lagged effects analyses provided some evidence that response delays were associated with activity behaviors occurring before an EMA prompt, the effect sizes of lagged effects of these behaviors dwindled quickly within just a few minutes before a prompt. This suggests that attempts to predict when respondents may be most responsive to EMA prompts based on passive activity sensors may be most successful when considering gradual and transient changes in physical activity levels.

As hypothesized, we found that respondents’ activity levels changed during response delays. Specifically, participants appeared to transition from either light, moderate, or vigorous activities to less active (standing or sedentary) states that were similar to activity levels for prompts that were *not* delayed. The duration of the response delay did not significantly moderate the amount of change in activities, suggesting that respondents may have tended to postpone responding until they were in a less active state, regardless of how long it took them to reduce their activity levels.

However, analyses of participants’ EMA ratings did not show that this change in objective activity levels systematically affected self-reported activity levels either for participants who were asked about the 5 min or about the 2 h before the prompt. In line with prior research (e.g., Lim et al., 2015; Prince et al., 2020; Quinlan et al., 2021), participants tended to generally underreport sedentary time and to overreport the amount of physically active time in EMA compared to objective measurements of the same behaviors before the prompts. These differences were not, however, moderated by response delays, such that the EMA responses did not differ from what would be expected had the responses not been delayed. That is, delayed responses did not lead to a selective representation of moments of high (or low) physical activity levels in the collected EMA data.

It is noteworthy that the present study explicitly asked respondents to recall and report their activity levels during the minutes “before the prompt.” An alternative instruction frequently used in EMA studies is to ask participants to report their “current” experiences and behaviors (Hall et al., 2021). Even though the latter instruction arguably minimizes potential recall biases compared with the former (Singh and Björling, 2019), it might have yielded a more pronounced impact of response delays on the self-reported activity levels as respondents’ current objective state (i.e., activity level) differed from that at the time of the prompt. However, this possibility remains speculative and will likely depend on how respondents interpret specific EMA instructions. For example, a previous study employed brief telephone-based cognitive interviews several times a day at random intervals to investigate which time periods EMA respondents would take into account for their momentary ratings (Wen et al., 2021). Results showed that unless participants were specifically trained to focus on the time right before the telephone call, only about half of the participants closely adhered to the time period as intended by the instructions. The extent to which EMA instructions modulate the impact of delayed responding will need to be empirically tested in future (experimental and qualitative interview) studies.

Examining the correlations between objective and EMA reported physical activities, we found evidence to suggest that delayed EMA reporting was associated with more random errors in measurement when respondents were asked about the 5 min before the prompt. Immediate EMA reports showed moderate correlations between objective and EMA reported activity data, corresponding with prior research (Knell et al., 2017; Stinson et al., 2022), whereas these correlations were significantly attenuated for delayed EMA reports. That said, the effect sizes for the differences in correlations were generally small, suggesting that potential measurement errors resulting from delayed responses are not likely to markedly reduce the quality and precision of the overall EMA data collected, especially when the rate of delayed responses is modest. However, this finding is consistent with the idea that memories of past events and behaviors fade quickly over time and that imprecisions in the recall of behaviors occur after very few minutes of delay time (Conner and Barrett, 2012). It is important to keep in mind that the maximum allowable delay in the present study was 15 min, and that EMA studies vary widely in the maximum response delay and have allowed response latencies of 30 min or longer (Eisele et al., 2021). Even though the present results cannot confirm this, it is possible that response delays of more than 15 min would be associated with even more pronounced reporting errors.

In secondary analyses conducted with EMA items that asked about activity levels over 2 h before the prompt rather than examining the shorter 5-min reporting period, we found very little evidence that delayed EMA responding was associated with attenuated correlations between objective and self-reported activities. Possible advantages and disadvantages of using EMA items with longer reporting periods such as the “past 2 h” (the so-called “coverage” model of EMA) have been discussed in Stone et al. (2023a). As suggested by the present results, a notable feature of longer reporting periods is that they are likely robust to potential reporting errors when individuals need to delay prompt responses, especially if the length of delays is brief (e.g., up to 15 min) relative to the time period covered by the EMA report (e.g., past 2 h). However, we acknowledge that this advantage may be offset if participants struggle to remember the entire time period covered by the EMA question, such that memory heuristics (e.g., peak and end heuristics) that are unrelated to the occurrence of response delays may come into play.

When deciding on the maximum allowable response delay in an EMA study, researchers may ask whether setting delayed responses to missing values would help reduce potentially biased estimates. Our results suggest that allowing for response delays of 15 min or less may not substantially bias EMA data on self-reported physical activity. At the same time, because respondents systematically delayed responses when they were more active, *not* collecting delayed responses (or discarding them from the data) could come at the cost of introducing missing values that are not missing (completely) at random, which could bias study results (Stone et al., 2023a).

### Limitations

The study sample was drawn from a larger general US population sample of internet panelists who regularly participated in online surveys. Prior research has shown that the samples recruited from these panels are not necessarily representative of the general population (Stone et al., 2023b) and the observed effects may be different in other (clinical) populations which may differ in the patterns of delayed EMA responses and in their motivation for timely assessment completion. As EMA is increasingly applied in clinical populations, further research on the occurrence and impact of delayed responses in clinical samples is needed.

Our study exclusively focused on physical activity behaviors, and the results may not necessarily translate to EMA reports of other behaviors or internal states. Focusing on physical activities provided a near-unique opportunity to compare EMA reports with continuous real-time recordings of the same (or closely related) concept. With few notable exceptions, constructs typically evaluated with EMA lack objective equivalents. For instance, health behaviors such as smoking and alcohol use that can be indirectly monitored in ambulatory settings (McClure et al., 2018), and environmental features such as noise levels can be passively recorded in real-time (Timmer et al., 2017). Moreover, biological correlates of psychological states such as stress have been inferred from ambulatory physiological data (e.g., skin conductance, heart rate variability), even though a close approximation of internal states remains challenging (Smets et al., 2018).

This study is limited by the use of the activPAL device, which primarily captures lower-body movements. Moderate or vigorous physical activities involving upper-body movements or minimal lower-body engagement, such as weightlifting or rowing, may have been missed as they are not fully captured by this thigh-worn device. In addition, the resolution of accelerometry-derived physical activities was 1 min, which may have introduced error variance due to some uncertainty about activity levels at the exact time of an EMA prompt.

The present study was also limited to two specific EMA designs that differed in the time period targeted in the EMA reports. Multiple characteristics of the prompting schedule (prompt frequency, number and timing of follow-up alarms, maximum allowable delay time) may act in concert to shape the occurrence and duration of delayed EMA responses, and differences in the design of EMA questions (time period covered by EMA questions, instructions about reporting the moments before the prompt or the current moments) may affect how response delays impact the precision and accuracy of EMA reports. Future studies are needed to investigate the occurrence and impact of response delays for various study design features.

## Conclusion

Delayed responses are a frequently overlooked “subtle” form of noncompliance in EMA studies. Participants in this study were more likely to delay EMA responses when they were more physically active, and responded after activity levels were significantly lower. Brief response delays did not systematically bias the physical activity levels captured with EMA, although they were associated with more random errors in EMA reports, with generally small effect sizes. To empirically inform best practices regarding the treatment of delayed responses in EMA studies, further observational and experimental research should investigate the generalizability of effects of delayed EMA responses across populations and EMA contents. Investigations in clinical populations are warranted considering that patients may differ from nonpatients in the frequency and reasons for response delays (such as physical symptoms that impede timely assessment completion). In addition, examining the effects of delayed responses across EMA contents may be especially important considering that concrete behaviors may be more memorable and less impacted by response delays compared to internal states that may be most accessible in the moments they are experienced. Finally, studies should systematically compare different features of EMA study designs, for example, to examine whether certain instructions for EMA reporting (“right now” versus “before the prompt”) are more susceptible to biases from delayed responses, and to investigate the quality of EMA responses that are substantially delayed (e.g., by more than 15 min).

## Data Availability

The data are not publicly available, but can be shared upon request. Requests to access the data should be directed to AS, arthuras@usc.edu.

## Footnotes

1 Objective physical activity levels recorded for the 5 min before the prompt were analyzed here in accordance with the time period the EMA reports in the primary analyses were supposed to capture. For the secondary analyses of respondents who received the 2-h version of EMA items, objective physical activity levels for the 120 min before the prompt were analyzed.

2 The intraclass correlation was calculated as τ / (τ + 3.29), where τ represents the estimated between-person variance of in a multilevel logistic regression model with no predictor variables, and 3.29 is the fixed within-person variance of a standard logistic distribution.
