# Supplementary material for: Do delayed responses introduce bias in ecological momentary assessment? Evidence from comparisons between self-reported and objective physical activity
Source: Front Psychol. 2025 Jan 3;15:1503411. doi: 10.3389/fpsyg.2024.1503411 (PMC11739121; doi:10.3389/fpsyg.2024.1503411)
Supplement: Supplementary file 1 [file Table_1.docx]

**Supplemental Online Materials**

**Do delayed responses introduce bias in Ecological Momentary Assessment? Evidence from comparisons between self-reported and objective physical activity**

Schneider, S., Toledo, M.J., Junghaenel, D.U., Smyth, J.M., Lee, P.J., Goldstein, S., Pomeroy, O., Stone, A.A.S.

Table S1: Missing responses predicted from objective measures of physical activity recorded during the minute of the EMA prompt in multilevel logistic regression models.

| Parameter | Sedentary | Standing | LPA | MPA | VPA | Steps |
| --- | --- | --- | --- | --- | --- | --- |
| Fixed effects |  |  |  |  |  |  |
| Intercept | -2.506 (.34)*** | -2.966 (.18)*** | -2.899 (.15)*** | -2.945 (.09)*** | -2.843 (.08)*** | -3.008 (.15)*** |
| Within-person | -.058 (.02)***  OR = 0.94 | .037 (.02)*  OR = 1.04 | .072 (.03)*  OR = 1.08 | .167 (.05)***  OR = 1.18 | .362 (.14)*  OR = 1.44 | .072 (.02)***  OR = 1.08 |
| Between-person | -.089 (.08)  OR = 0.91 | .082 (.11)  OR = 1.08 | .126 (.28)  OR = 1.13 | .945 (.50)*  OR = 2.57 | .239 (2.00)  OR = 1.27 | .209 (.16)  OR = 1.23 |
| Variance components |  |  |  |  |  |  |
| Intercept | 1.193 | 1.176 | 1.183 | 1.161 | 1.185 | 1.174 |
| Within-person   regression slope | .054 | .015 | .016 | .000 | .069 | .010 |
| Covariance | .013 | -.050 | -.095 | .013 | -.305 | .001 |

Note: Standard errors are in parentheses. * *p* < .05; *** *p* < .001. LPA = light physical activity; MPA = moderate physical activity; VPA = vigorous physical activity; OR = odds ratio. Predictors are scaled in 10 seconds/minute (for time sedentary, time standing, time in LPA, time in MVPA) or in 10 steps/minute (for step count).

Table S2: Results from time-to-event (Cox proportional hazard) models predicting the occurrence of EMA responses over time from objective measures of physical activity recorded during the minute of the EMA prompt.

| Parameter | Sedentary | Standing | LPA | MPA | VPA | Steps |
| --- | --- | --- | --- | --- | --- | --- |
| Fixed effects |  |  |  |  |  |  |
| Within-person   estimate | .062 (.01)***  HR = 1.06 | -.045 (.01)***  HR = 0.96 | -.136 (.01)***  HR = 0.87 | -.124 (.02)***  HR = 0.88 | -.263 (.06)***  HR = 0.77 | -.097 (.01)***  HR = 0.91 |
| Between-person  estimate | .169 (.04)***  HR = 1.18 | -.163 (.04)***  HR = 0.85 | -.480 (.11)***  HR = 0.62 | -.814 (.23)***  HR = 0.44 | -.115 (.89)  HR = 0.89 | -.324 (.07)***  HR = 0.72 |
| Random effect |  |  |  |  |  |  |
| Frailty variance | .310 | .306 | .308 | .302 | .310 | .311 |

Note: Standard errors are in parentheses. *** *p* < .001. LPA = light physical activity; MPA = moderate physical activity; VPA = vigorous physical activity; HR = hazard ratio. Predictors are scaled in 10 seconds/minute (for time sedentary, time standing, time in LPA, time in MVPA) or in 10 steps/minute (for step count).

Table S3: Change in objective physical activity levels from the minute of the prompt to the minute of the response for delayed EMA responses, moderated by the duration of the delay

| Parameter | Sedentary (seconds) | Standing (seconds) | LPA  (seconds) | MPA  (seconds) | VPA  (seconds) | Steps  (count) |
| --- | --- | --- | --- | --- | --- | --- |
| Fixed effects |  |  |  |  |  |  |
| Intercept (time of   prompt) | 32.12 (.99)*** | 18.94 (.77)*** | 7.12 (.44)*** | 1.47 (.22)*** | 0.33 (.11)* | 11.94 (.77)** |
| Change from prompt   to response | 2.13 (1.07)* | .80 (.92) | -2.23 (.49)*** | -0.42 (.24) | -0.33(.11)* | -4.25 (.81)*** |
| Delay duration | 0.29 (.74) | -0.05 (.61) | -0.38 (.36) | -0.05 (.18) | 0.17 (.09) | -0.20 (.60) |
| Change × delay   duration | -1.16 (.78) | 0.98 (.69) | 0.35 (.39) | 0.04 (.20) | -0.17 (.09) | 0.07 (.66) |
| Variance components |  |  |  |  |  |  |
| Level 2 random effects |  |  |  |  |  |  |
| Intercept | 59.54 | 22.26 | 4.62 | 1.58 | 0.05 | 26.11 |
| Change | 83.27 | 48.34 | 7.62 | 0.63 | 0.00 | 17.59 |
| Covariance | -24.32 | -7.73 | -2.84 | -0.63 | -0.02 | -13.88 |
| Level 1 residuals |  |  |  |  |  |  |
| Time of prompt | 677.16 | 526.69 | 85.31 | 27.54 | 0.02 | 219.37 |
| Time or response | 745.05 | 522.98 | 181.80 | 44.66 | 13.38 | 503.00 |
| Covariance | 307.01 | 197.51 | 25.72 | 6.97 | -0.02 | 55.23 |

Note: Standard errors are in parentheses. **p* < .05, ***p* < .01, ****p* < .001. LPA = light physical activity; MPA = moderate physical activity; VPA = vigorous physical activity.

Table S4: Results of multilevel models comparing objective and EMA reported physical activity levels for the 120 minutes before the prompt across immediate and delayed responses

| Parameter (scaled in seconds per minute) | Sedentary | Standing | LPA | MPA | VPA |
| --- | --- | --- | --- | --- | --- |
| Fixed effects |  |  |  |  |  |
| Intercept | 37.14 (.54)*** | 12.86 (.40)*** | 9.24 (.28)*** | 1.68 (.11)*** | .65 (.10)*** |
| Assessment type | -5.44 (.62)*** | -3.00 (.58)*** | 8.92 (.44)*** | 1.29 (.19)*** | .82 (.09)*** |
| Response delay | -2.53 (.65)*** | 1.05 (.84)*** | 1.17 (.35)*** | .53 (.19)** | .21 (.07)** |
| Assessment type × delay | -1.12 (.70) | .04 (.61) | 1.00 (.60) | .53 (.33) | .08 (.10) |
| Variance components |  |  |  |  |  |
| Level 2 random effects |  |  |  |  |  |
| Intercept | 41.51 | 22.83 | 11.10 | 1.46 | 0.38 |
| Assessment type | 56.19 | 51.13 | 26.32 | 4.13 | 1.24 |
| Response delay | 0.00 ^a^ | 0.67 | 0.00 | 0.26 | 0.02 |
| Assessment type × delay | 9.10 | 4.75 | 4.10 | 0.61 | 0.00 ^a^ |
| Level 1 residuals |  |  |  |  |  |
| Accelerometry assessments | 196.17 | 116.20 | 16.20 | 8.00 | 2.28 |
| EMA assessments | 298.24 | 147.06 | 178.78 | 48.60 | 4.07 |
| Covariance | 152.24 | 56.17 | 19.88 | 4.48 | 0.72 |

Note: ** *p* < .01; *** *p* < .001. Assessment type = EMA reports versus objective recordings. Response delay = delayed versus immediate EMA responses. ^a^ Variance components were fixed at zero to achieve a positive definite covariance matrix of the random effects. LPA = light physical activity; MPA = moderate physical activity; VPA = vigorous physical activity

Table S5: Multilevel regression models predicting objective physical activity levels during the 120 minutes before the EMA prompt from EMA reported physical activity levels, for immediate versus delayed EMA responses

|  | Physical activity outcomes for EMA items with open numeric response format | | | | |  | Physical activity outcomes for EMA items with rating scale format^a^ | |
| --- | --- | --- | --- | --- | --- | --- | --- | --- |
| Parameter (scaled in seconds per minute) | Sedentary | Standing | LPA | MPA | VPA ^b^ |  | Sedentary | LMVPA |
| Fixed effects |  |  |  |  |  |  |  |  |
| Intercept | 40.44 (.56)*** | 13.70 (.42)*** | 4.69 (.16)*** | 1.06 (.10)*** | .44 (.13) |  | 40.94 (.56)*** | 5.60 (.21)*** |
| Delay | -.48 (.60) | .83 (.54) | .53 (.22)* | .20 (.16) | -.04 (.06) |  | -.68 (.67) | .26 (.26) |
| EMA | .52 (.02)*** | .40 (.02)*** | .12 (.01)*** | .10 (.02)*** | .05 (.02) |  | 7.63 (.35)*** | 3.45 (.20)*** |
| Delay × EMA | .05 (.04) | .01 (.05) | -.04 (.02)* | -.01 (.02) | -.02 (.02) |  | -.39 (.66) | .00 (.29) |
| Random effects |  |  |  |  |  |  |  |  |
| Intercept | 46.31 | 24.30 | 3.72 | 1.21 | 2.74 |  | 45.98 | 6.19 |
| EMA regression   slope | .03 | .03 | .01 | .04 | .05 |  | 12.36 | 4.77 |
| Covariance | -.64 | .11 | .05 | .15 | .36 |  | -8.58 | 4.29 |
| Level 1 residual | 95.33 | 82.20 | 12.97 | 6.89 | .79 |  | 112.89 | 17.30 |

Note: **p* < .05, ** *p* < .01, *** *p* < .001. LPA = light physical activity; MPA = moderate physical activity; VPA = vigorous physical activity; LMVPA = light, moderate, or vigorous physical activity. ^a^ EMA rating scale items were “during the 5 minutes before the prompt, how sedentary were you?” and “during the 5 minutes before the prompt, how physically active were you?” ^b^ accelerometry and EMA data for VPA were log transformed to facilitate model convergence.

Table S6: Within-subject correlations between objective physical activity levels during the 120 minutes before the EMA prompt and EMA reported physical activity levels, for immediate and delayed EMA responses

|  | Correlation between objective and EMA self-reported activities | |  | Difference in correlations |
| --- | --- | --- | --- | --- |
| Physical activity variable | Immediate response | Delayed response |  | Effect size q |
| Time sedentary | .63 | .65 |  | -03 |
| Time standing | .38 | .43 |  | -.06 |
| Time in LPA | .38 | .27 |  | .12 |
| Time in MPA | .23 | .17 |  | .06 |
| Time in VPA | .24 | .23 |  | .01 |
| Rating of how sedentary | .54 | .51 |  | .04 |
| Rating of how physically active | .57 | .56 |  | .01 |
